# Association of T-wave electrocardiogram changes and type 2 diabetes: a cross-sectional sub-analysis of the MASHAD cohort population using the Minnesota coding system

**DOI:** 10.1186/s12872-023-03649-2

**Published:** 2024-01-13

**Authors:** Sara Soflaei Saffar, Eisa Nazar, Toktam Sahranavard, Farzad Fayedeh, AmirAli Moodi Ghalibaf, Mahmoud Ebrahimi, Hedieh Alimi, Bahram Shahri, Azadeh Izadi-Moud, Gordon A. Ferns, Alireza Ghodsi, Saeed Mehrabi, Milad Tarhimi, Habibollah Esmaily, Mohsen Moohebati, Majid Ghayour-Mobarhan

**Affiliations:** 1 International UNESCO Center for Health-Related Basic Sciences and Human Nutrition, Mashhad University of Medical Sciences, Mashhad, Iran; 2 Orthopedic Research Center, Mazandaran University of Medical Sciences, Sari, Iran; 3 Student Research Committee, Birjand University of Medical Sciences, Birjand, Iran; 4 Vascular and Endovascular Research Center, Faculty of Medicine, Mashhad University of Medical Sciences, Mashhad, Iran; 5 Department of Cardiology, Faculty of Medicine, Mashhad University of Medical Sciences, Mashhad, Iran; 6 Brighton and Sussex Medical School, Division of Medical Education, Brighton, UK; 7 Department of Cardiology, Faculty of Medicine, Gonabad University of Medical Sciences, Mashhad, Iran; 8 Department of Biostatistics, School of Health, Mashhad University of Medical Sciences, Mashhad, Iran; 9 Social Determinants of Health Research Center, Mashhad University of Medical Sciences, Mashhad, Iran; 10 Metabolic Syndrome Research Center, Mashhad University of Medical Sciences, Mashhad, 99199-91766 Iran; 11 Psychiatry and Behavioral Sciences Research Center, Addiction Institute, Mazandaran University of Medical Sciences, Sari, Iran

**Keywords:** Electrocardiogram, Type 2 diabetes mellitus, T-wave

## Abstract

**Background:**

Type 2 Diabetes Mellitus (T2DM) has become a major health concern with an increasing prevalence and is now one of the leading attributable causes of death globally. T2DM and cardiovascular disease are strongly associated and T2DM is an important independent risk factor for ischemic heart disease. T-wave abnormalities (TWA) on electrocardiogram (ECG) can indicate several pathologies including ischemia. In this study, we aimed to investigate the association between T2DM and T-wave changes using the Minnesota coding system.

**Methods:**

A cross-sectional study was conducted on the MASHAD cohort study population. All participants of the cohort population were enrolled in the study. 12-lead ECG and Minnesota coding system (codes 5–1 to 5–4) were utilized for T-wave observation and interpretation. Regression models were used for the final evaluation with a level of significance being considered at *p* < 0.05.

**Results:**

A total of 9035 participants aged 35–65 years old were included in the study, of whom 1273 were diabetic. The prevalence of code 5–2, 5–3, major and minor TWA were significantly higher in diabetics (*p* < 0.05). However, following adjustment for age, gender, and hypertension, the presence of TWAs was not significantly associated with T2DM (*p* > 0.05). Hypertension, age, and body mass index were significantly associated with T2DM (*p* < 0.05).

**Conclusions:**

Although some T-wave abnormalities were more frequent in diabetics, they were not statistically associated with the presence of T2DM in our study.

## Introduction

Type 2 Diabetes Mellitus (T2DM) is a complex condition associated with impaired glucose tolerance, insulin resistance and hyperglycemia; its increasing prevalence has become a serious global health challenge. It is accountable for 11.3% of deaths worldwide and is believed to affect approximately 10.9% of the global population [1]. T2DM is accompanied by debilitating chronic complications such as kidney disease, retinopathy, neuropathy, microvascular impairment, and cardiovascular complications [1, 2].

Cardiovascular complications are responsible for up to 68% of all diabetes-related mortalities. Several studies have revealed that patients with T2DM are at increased risk of coronary disease [3], myocardial infarction [4], heart failure [5], cardiomyopathy [6], and thrombotic events [7]. It has been shown that diabetic patients have a two- to three-fold increase in cardiovascular disease (CVD) development [8]. Various mechanisms have been proposed to explain the increased CVD rates among diabetic patients. Higher incidence of dyslipidemia [9], chronic inflammatory states [10–12], enhanced oxidative stress and reactive oxygen species [13], and hypercoagulability [14] are some of the key findings in patients with T2DM that can potentially increase atherosclerosis, plaque formation, and consequently result in increased rates of CVD [10, 15]. Thus, it is of great importance to investigate sufficient early detection methods and effective therapeutic approaches for CVD among diabetic patients.

An electrocardiogram (ECG) is a useful and non-invasive assessment that has been utilized for several biomedical uses such as determination of arrhythmias, fibrillations, heart rates, premature contractions, and ischemia [16–19]. T-wave in ECG represents ventricular repolarization. T-wave abnormalities (TWA) can be an indicator of a variety of conditions such as cardiomyopathy, pulmonary embolism, peri- and myocarditis, and ischemia [20–23].

Given the importance of T2DM and its complications, especially those affecting the cardiovascular system, as well as considering the ease of accessibility and practicality of ECG in medical practice, this cross-section study was designed to investigate the prevalence of T-wave abnormalities and its association with T2DM.

## Method

### Study design and participants

The current cross-sectional study was conducted on the population of Mashhad stroke and heart atherosclerotic disorder (MASHAD) cohort study [24]. A total of 9704 individuals aged 35 to 65 years were enrolled into this cohort study. A checklist containing participants’ demographic data including age, sex, educational level, and marital status was recorded. Patients whose systolic blood pressure levels were at or above 140 mmHg and/or diastolic blood pressure were at or beyond 90 mmHg—measured using a mercury sphygmomanometer- were considered hypertensive [25]. A fasting blood glucose (FBG) > 126 mg/dl, or being under anti-hyperglycemic medication was defined as diabetic patients [26]. The FBG was measured in a peripheral blood sample following 14 h of fasting [27]. The study was approved by the Human Research ethics committee of Mashhad University of Medical Sciences, Mashhad, Iran, and all participants provided informed consent prior to data collection.

### ECG analysis

A standard resting 12-lead ECG was taken from each participant of the study. These ECGs were interpreted by instructed medical students in accordance with Minnesota coding system [28]. Five percent of all ECGs were also read by certified cardiologists. Among the 9704 participants, the ECGs of 9035 participants were available and readable according to Minnesota coding system [28].

Four different t-wave abnormalities were described within the coding system including codes 5–1, 5–2, 5–3 and 5–4. The code 5–1 was defined as T amplitude negative 5.0 mm or more in either of leads I, V6, or in lead aVL when R amplitude is ≥ 5.0 mm and code 5–2 was defined as T amplitude negative or diphasic (positive–negative or negative–positive type) with negative phase at least 1.0 mm but not as deep as 5.0 mm in lead I or V6, or in lead aVL when R amplitude is ≥ 5.0 mm. Code 5–3 was described as flat, negative or diphasic t-wave with less than 1 mm negative phase in any leads of I, II or V3 to V6 or in lead aVL when the R amplitude is ≥ 5.0 mm. Lastly, code 5–4 was defined as a positive T amplitude and a T/R amplitude ratio < 1:20 in any of leads I, II, aVL, or V3 through V6. The R-wave amplitude must be ≥ 10.0 mm [28].

### Statistical analysis

Qualitative and quantitative variables were summarized as Mean $\pm$ SD and frequency (%), respectively. An independent t-test was used in order to compare the mean of quantitative variables between the two groups. In addition, evaluating the association between qualitative variables was performed using Chi-square and Fisher's exact test. Further analyses were performed in order to investigate the association between T wave impairments and T2DM after adjusting the effect of potential confounders (variables with *P* < 0.25 in the univariate logistic regression model) using the multiple logistic regression (LR) model. Furthermore, receiver operating characteristic (ROC) curves were used to evaluate the ability of the multiple LR model to predict the occurrence of TWA and T2DM. All statistical analyses were carried out using SPSS version 20 and the statistical significance level was considered at 0.05.

## Results

### Study population characteristics

**Fig. 1:**
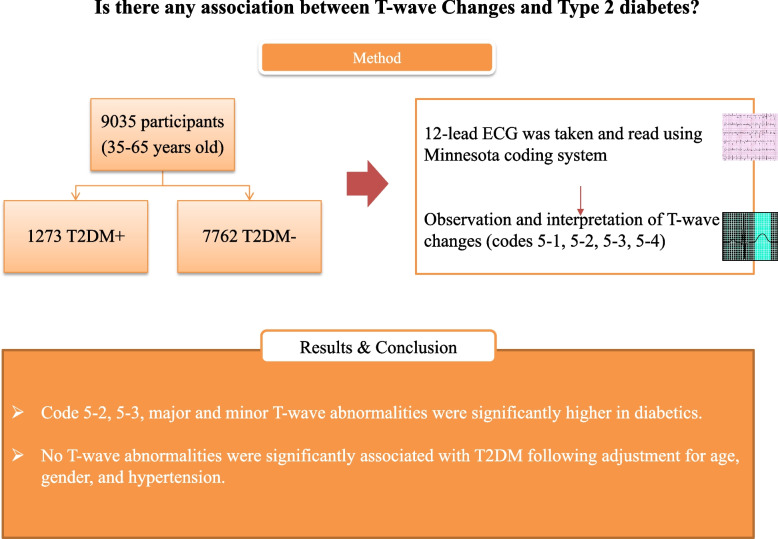
The summary of the methodology and the findings

A total of 9035 subjects were included into this study, including 1273 diabetic patients and 7762 non-diabetic individuals (Fig. 1). The average age was 47.45 ± 8.17 years and 51.77 ± 7.73 years in non-diabetic and diabetic patients which differed significant (*p* < 0.001). Diabetic patients were found to have higher body mass index (BMI), as well as higher rates of hypertension (50.3 vs 27.9%, *p* < 0.001). Marital status and educational levels also showed a significant different distribution between the two diabetic and non-diabetic groups with married being the most prevalent status among studied groups (*P* < 0.001). Table 1 presents patients’ demographic data distributions.

**Table 1:** Demographic and clinical characteristics of patients under study according to T2DM^a^

Variables^a^	**Total**	T2DM		*P*-value
		Yes (*N* = 1273)	No (*N* = 7762)	
Age (y)	51.77 ± 7.73	47.45 $\pm$ 8.17	51.77 $\pm$ 7.73	< 0.001^*^
Body mass index (kg/m^2^)	28.93 ± 4.62	27.71 $\pm$ 4.73	28.93 $\pm$ 4.62	< 0.001^*^
Gender				
Male	3615 (40.00)	3129 (40.30)	486 (38.20)	0.15
Female	5420 (60.00)	4633 (59.70)	787 (61.80)	
Marital status				
Single	55 (0.60)	51 (0.70)	4 (0.30)	< 0.001^*^
Married	8418 (93.20)	7262 (93.60)	1156 (90.80)	
Divorced/widowed	562 (6.20)	449 (5.80)	113 (8.90)	
Education level				
Illiterate	1155 (12.80)	927 (11.90)	228 (17.90)	< 0.001^*^
Lower than diploma	6815 (75.40)	5881 (75.80)	934 (73.40)	
Higher than diploma	1065 (11.80)	954 (12.30)	111 (8.70)	
Hypertension				
No	6220 (69.00)	5589 (72.10)	631 (49.70)	< 0.001^*^
yes	2798 (31.00)	2159 (27.90)	639 (50.30)	

^*^Significance level of 0.05

^a^Values are reported as Mean $\pm$ SD and frequency (%)

### T-wave abnormality frequency

**Fig. 2:**
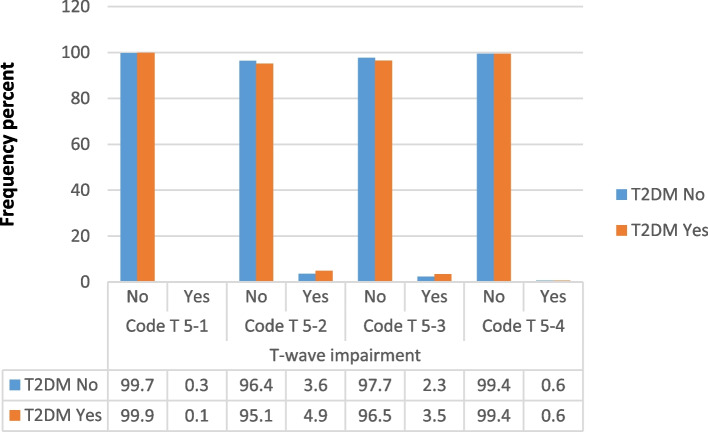
Comparison of frequency distribution of the T wave impairments between patients with and without T2DM (*n* = 9035)

**Fig. 3:**
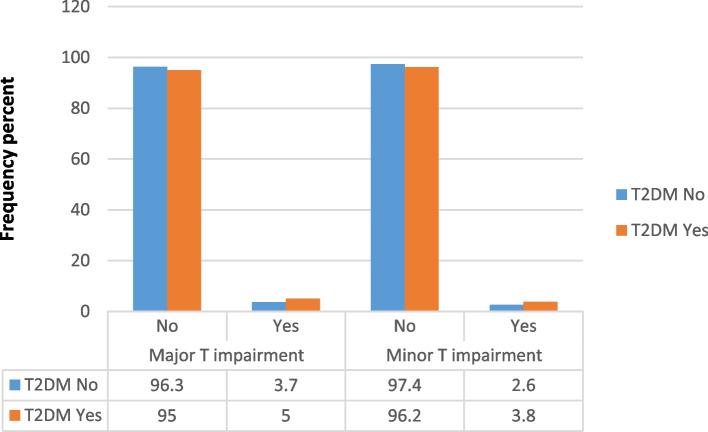
compares major T impairment and major T impairment between patients with and without T2DM (*n* = 9035)

A total of 1246 T wave abnormalities were reported among the study sample population, approximately 13.79% of all participants. The most frequent TWA among both groups were code 5–2 (4.9% in diabetics and 3.6% in the control group) and major T-wave abnormalities (5% in diabetics and 3.7% in the control group). Different TWA yielded varying associations with T2DM. While T-wave abnormalities code 5–1 and 5–4 failed to show a significantly different distribution among diabetic and non-diabetic participants (*P* = 0.24 and 0.92 respectively), code 5–2 and 5–3 were shown to be significantly higher among diabetic patients compared to the non-diabetic individuals (*P* = 0.02 and 0.01, respectively). Overall, both major and minor T-wave abnormalities were significantly more frequent among patients with T2DM compared to the control group, (*p* = 0.02 and 0.008, respectively). Figures 2 and 3 compare T wave impairments and T2DM distribution.

### T2DM predictive factors

**Fig. 4:**
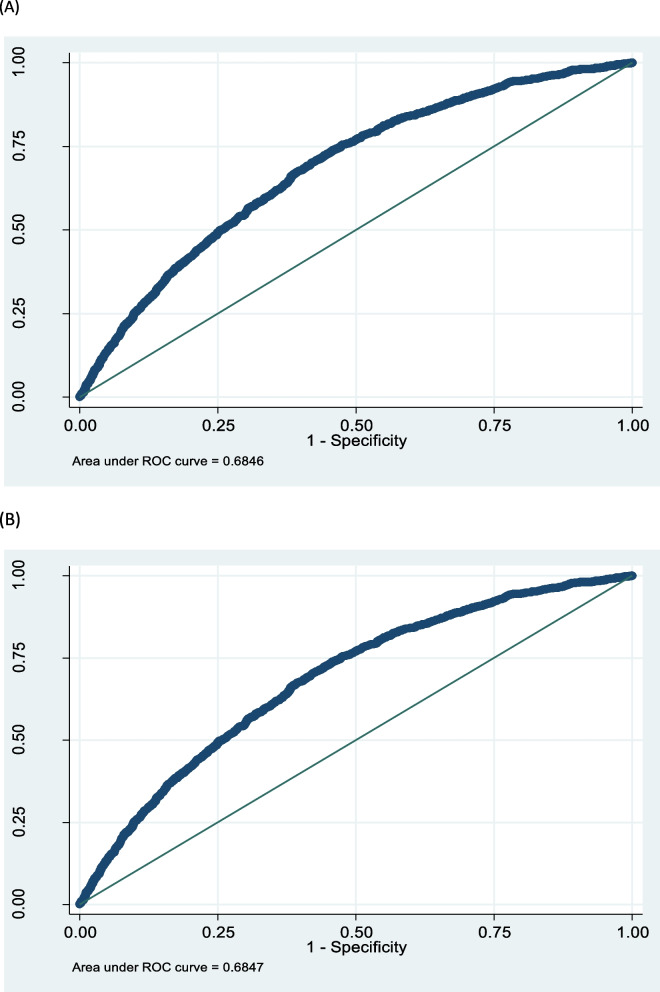
**A** showing the predictive power of final multiple LR model including T-wave abnormality code 5–2 and 5–3 to predict diabetes and **B** showing the predictive power of final multiple LR model including major and minor T wave abnormality to predict diabetes

Results from the multiple logistic regression models indicated a significant association between age (OR = 1.05, 95%CI = 1.04–1.05) and BMI (OR = 1.03, 95%CI = 1.02–1.05) with having T2DM. Gender, marital status, and educational level did not show a significant association with having T2DM (all *P* > 0.05) (Tables 2 and 3). Hypertension has been reported to increase the odds of T2DM by 86 percent (95%CI = 1.63–2.12, *p* < 0.001). According to Tables 2 and 3, only major and minor T wave impairments as well as impairments code 5–2 and 5–3 were reported to be higher among diabetic patients and thus only these items were further analyzed by inclusion in the multiple LR model. A model analyzing T-wave abnormality code 5–2 and 5–3 showed that the odds of having T2DM among patients with T-wave code 5–2 and 5–3 abnormalities were 1.07 and 1.31 times as those without these abnormalities, respectively. This observed difference between patients with and without T-wave abnormalities regarding having T2DM failed to yield statistical significance (*P* = 0.63 and 0.12, respectively). The area under the ROC curve (AUC) of the final multiple LR model was 0.6847, which indicates a good predictive power of the final model, as shown in Fig. 4A. Also, the results of our model for major and minor T-wave abnormality revealed that the odds of having T2DM in patients who had T major and T minor abnormalities were 1.06 and 1.30 times than those without ischemia abnormalities, respectively. However, this difference did not show a significant difference within the logistic regression model (*P* = 0.65 and 0.11 respectively). The AUC for this model was 0.6846 which suggests a good predictive power of the final model, as shown in Fig. 4B. Tables 2 and 3 presents the results of the regression model analyses.

**Table 2:** Investigating the association between T-wave impairment and having diabetes adjusted by age, BMI, gender, education, and marital status using multiple LR model

Variables	OR^a^	95% CI	*P*-value
Age	1.05	1.04- 1.05	< 0.001^*^
BMI	1.03	1.02—1.05	< 0.001^*^
Gender			
Female	Ref	Ref	0.88
Male	1.01	0.88—1.16	
Marital status			
Single	Ref	Ref	-
Married	1.20	0.42—3.37	0.72
Divorced/Widowed	1.34	0.46—3.85	0.58
Educational level			
Illiterate	Ref	Ref	-
Lower than diploma	0.94	0.79—1.11	0.48
Higher than diploma	0.73	0.56—0.95	0.02^*^
Hypertension			
No	Ref	Ref	< 0.001^*^
Yes	1.86	1.63—2.12	
Code 5–2 T wave impairment			
No	Ref	Ref	0.63
Yes	1.07	0.80—1.44	
Code 5–3 T wave impairment			
No	Ref	Ref	0.12
Yes	1.31	0.92—1.87	

^*^Significance level of 0.05

^a^*OR* Odds Ratio

**Table 3:** Investigating the association between major T-wave impairment and minor T-wave impairment with having diabetes adjusted by age, BMI, gender, education, and marital status using multiple LR model

Variables	OR^a^	95% CI	*P*-value
Age	1.05	1.04- 1.06	< 0.001^*^
BMI	1.03	1.02—1.05	< 0.001^*^
Gender			
Female	Ref	Ref	0.88
Male	1.01	0.88—1.16	
Marital status			
Single	Ref	Ref	-
Married	1.20	0.42—3.37	0.72
Divorced/Widowed	1.34	0.46—3.84	0.58
Educational level			
Illiterate	Ref	Ref	-
Lower than diploma	0.94	0.79—1.11	0.48
Higher than diploma	0.73	0.56—0.95	0.02^*^
Hypertension			
No	Ref	Ref	< 0.001^*^
Yes	1.86	1.63—2.12	
Major T-wave impairment			
No	Ref	Ref	0.65
Yes	1.06	0.79—1.42	
Minor T-wave impairment			
No	Ref	Ref	0.11
Yes	1.30	0.93—1.82	

^*^Significance level of 0.05

^a^*OR* Odds Ratio

## Discussion

The current study aimed to investigate the distribution of t-wave impairment among diabetic patients and its association with T2DM according to the Minnesota coding system. The primary results showed significantly higher rates of code 5–2 and 5–3 t-wave impairment among diabetic patients. Both minor and major t-wave abnormalities were also significantly higher among diabetics. However, upon adjusting for several factors such as age, gender, and hypertension within the regression model, none of the mentioned t-wave abnormalities showed a significant association with T2DM.

Myocardial ischemia is a relatively frequent finding among diabetic patients and can potentially lead to coronary artery disease [29, 30]. Patients with myocardial ischemia can present both symptomatic and asymptomatic, with or without previous cardiovascular events. The rates of silent asymptomatic myocardial ischemia have been shown to be three to six times higher among diabetic patients [29]. Atherosclerosis and endothelial damage of vessels has been shown to be strong risk factors for ischemic heart disease (IHD). On the other hand, the formation of plaque and thrombi can lead to acute forms of myocardial ischemia and coronary syndromes [31, 32]. T2DM can contribute substantially to atherogenesis [33], thrombosis [34], and vascular damage [35], therefore leading to increased risks of IHD [36]. Hyperglycemia, increased levels of free fatty acids, and insulin resistance can lead to several destructive mechanisms such as inflammation, oxidative stress, and the production of advanced glycation products (AGE) [36, 37]. Following the increase in AGE production, inflammatory responses are triggered and pro-inflammatory transcription factors such as NF-kB are upregulated [38, 39]. Vascular motion is also affected via the reduction in nitric oxide synthesis and enhanced endothelin-1 release. Upregulated pro-thrombotic tissue factor and plasminogen activator inhibitor-1 levels, as well as decreased tissue plasminogen activator within T2DM, can lead to thrombi formation [36, 40]. The results of these various mechanisms is endothelial dysfunction, vasoconstriction, and enhanced plaque formations, which as mentioned before, are key components in the development and progression of IHD [36, 40].

Several studies have shown TWA among diabetic patients and their utilization as risk predictors. A 2021 study by Molud et al. studied the relationship between TWA and cardiovascular events among diabetic patients [41]. Minnesota code 5–1 and 5–2 were considered major TWA and codes 5–3 and 5–4 were considered to be minor TWAs. Their results indicated that patients with TWA had increased risks of both cardiovascular and all-cause mortality and major TWA was attributed to higher risk than minor TWAs [41]. They also highlight the usefulness of TWA in prognostication of diabetic patients in long-term settings. According to a prospective longitudinal study by Harms et al. [42] 45% of diabetic patients had or develop ECG abnormalities and 7.5% developed major adverse cardiac events within a 6.6-year follow-up period. Upon grading ECG abnormalities using the Minnesota coding system, 6 and 5% of the diabetic population had minor and major ST-segment/T-wave abnormalities respectively. They also concluded that ST-segment/T-wave abnormalities were associated with heart failure and coronary heart disease. Thus, T-T-wave modifications can be used as risk predictor for cardiovascular events and mortality among diabetic patients. In addition to ST/T-wave changes which exhibit ischemic disorders, signs of decreased conductivity such as PR and QRS prolongation, and hypertrophy such as tall R-wave was also observed in diabetics and were associated with chronic heart disease [42].

T-wave variation and abnormalities have also been shown within several other diabetes-related pathologies other than IHD. T-wave inversion within some diabetic patients can be explained via hyperkalemia. Diabetic ketoacidosis is a state of hyperkalemia and can result in a variety of ECG modifications affecting T-wave, QT, and ST segments [43]. T-wave inversion is also associated with left ventricle hypertrophy findings of ECG among diabetic patients, which might indicate myocardial injury but not coronary disease [44]. This finding is contradicted by another study, in which, ST-T changes are significant predictors of coronary artery disease, defined as elevated, depressed, or inversed T waves [45]. The observed difference can be due to sample size or ECG coding and grading system.

Some of the novel ECG parameters such as the QRS-T angle and T-wave axis of the frontal plane have also been investigated in diabetic patients by other studies [46]. It has been shown that 20.9% of diabetic patients have abnormal T-wave axis while 14% of them have increased QRS-T angle. These two ECG parameters are associated with some atherosclerotic disease markers among type II diabetic patients [46].

Studies on the relationship between T2DM and T-wave abnormalities have reported inconsistent results. A Chinese study investigated ECG abnormalities within several disorders such as hypertension, smoking, obesity, and so forth [47]. T2DM was found to be associated with ST elevation but failed to show a significant correlation with other electrocardiogram findings such as ST depression, T-wave and Q-wave impairment, tall R wave, atrial hypertrophy, and axial deviations. Unlike T2DM, hypertension, and hypercholesterolemia were significantly attributed to ST depression and T-wave abnormalities. These findings are in line with the results of our study, since upon adjustment, none of the T-wave abnormalities were associated with T2DM. However, two studies showed a contrary result. Flatter and asymmetric T-waves were observed in patients with type I diabetes, according to the study by Isaksen et al. [48]. This association was also confirmed by a regression model corrected for age, gender, BMI, blood pressure, potassium, and cholesterol. Interestingly, asymmetrical t-wave was significantly associated with both macro and microalbuminuria among type I diabetic patients. An Italian cross-sectional study [49] also confirmed this finding and suggests higher rates of T-wave axis abnormalities – described as T-wave rotation in the frontal plane – in diabetic patients compared to non-diabetic individuals [49]. These differences could be due to a lack of differentiating diabetes types, as well as the ethnicity of the study population.

Even though our analysis showed no significant association between T2DM and T-wave changes in the ECG, several other factors such as hypertension, age, and BMI were found to be significantly associated with T2DM. A meta-analysis of a total of 452,584 patients also showed similar results about the association between T2DM and hypertension (pooled OR:8.32, 95%CI: 3.05–22.71) [50]. The mechanisms by which, diabetes increases risks of hypertension can be explained through disturbed sodium homeostasis, insulin resistance, enhanced volume expansion and prominent resistance within peripheral vessels [51]. Our results indicated no significant relationship between gender and T2DM, whereas some studies show a significant contribution of sex and T2DM [52]. A longitudinal study in Iran showed significantly higher rates of T2DM among females while the global prevalence is higher in men [53, 54]. These differences in findings can be due to sampling size as well as not differentiating the type of diabetes among different studies. It has been also shown that gender differences poses varied risks of diabetes development among different races [55].

This study is one of very few studies to differentiate T-wave abnormalities into six categories, while most of the studies only summarize them in two. Second, a large population (*n* = 9035) was examined and observed in this cross-section study which belonged to the MASHAD cohort study. Third, some of the interpretations were also controlled by certified cardiologists which reduce the chances of errors. However, our study faced several limitations which need to be considered for future studies. First, available documentation did not differentiate type I or II diabetes, and thus, exact conclusions cannot be made for each type. Second, the age group of the study was limited to 35–65 years old, and variation might exist in ages above or below the cutoff used in our study. Third, only t-waves were used for ischemic changes of the heart, and future studies can use several other modalities, such as other ECG findings, and other para-clinical values to further confirm ischemic diseases of the heart due to T2DM. We also highly encourage future researchers to perform multi-central cohort studies in order to precisely evaluate the relationship between the two. High-quality meta-analyses are needed for confirming our findings.

## Conclusion

The results of this study show a significantly higher prevalence of Minnesota codes 5–2, 5–3, major and minor T-wave abnormalities in diabetic patients compared to non-diabetic individuals. However, the association between these abnormalities was not significant using regression models and adjusting for age, gender, and BMI. Considering the aberrant T2DM complications, especially cardiovascular ones, it is highly important to investigate CVD diagnostic tools among diabetics. Given the contrary results of other studies, large-scale studies on the topic of using t-wave abnormalities as ischemic pathologies resulting from T2DM are needed for further identification of sufficient indicative and predictive tools.

## Data Availability

The authors confirm that the data supporting the findings of this study are available from the corresponding author on request.

## Abbreviations

T2DM: Type 2 Diabetes Mellitus
TWA: T-wave abnormalities
ECG: Electrocardiogram
CVD: Cardiovascular disease
MASHAD: Mashhad stroke and heart atherosclerotic disorder
FBG: Fasting blood glucose
LR: Logistic regression
ROC: Receiver operating characteristic
BMI: Body mass index
AUC: Area under the ROC curve
IHD: Ischemic heart disease
AGE: Advanced glycation products
